# The TRAPP complexes: oligomeric exchange factors that activate the small GTPases Rab1 and Rab11

**DOI:** 10.1002/1873-3468.14553

**Published:** 2022-12-18

**Authors:** Antonio Galindo, Sean Munro

**Affiliations:** ^1^ MRC Laboratory of Molecular Biology Cambridge UK

**Keywords:** autophagy, exchange factor, Golgi apparatus, membrane traffic, Rab GTPase, recycling endosome

## Abstract

The Transport Protein Particle (TRAPP) complexes are highly conserved multisubunit complexes that act as nucleotide exchange factors (GEFs) for Rab GTPases. They act in both protein secretion and autophagy and have also been proposed to have a role in other processes such as cytokinesis and ciliogenesis. There are two TRAPP complexes in metazoans: TRAPPII, which activates Rab11; and TRAPPIII, which activates Rab1. Both complexes share a core of small subunits that form the active site for the exchange of GDP for GTP. In addition, each TRAPP complex has distinct large subunits that determine the specificity of each complex towards its substrate Rab and are essential for activity *in vivo*. Crystal structures have revealed the organisation of the TRAPP core and the mechanism of Rab1 activation, whilst recent cryo‐EM structures have unveiled the arrangement of the specific subunits around the core to form each complex. Combining these findings with functional experiments has allowed the proposal of mechanisms for how the specificity of each complex towards their cognate Rab is determined and for the arrangement of these large complexes on the membrane.

## Graphical abstract

TRAPPII and TRAPPIII are highly conserved multisubunit complexes that activate the small GTPases, Rab11 and Rab1, respectively. They act in protein secretion and autophagy and share a common active site. Each TRAPP complex has distinct subunits that determine their specificity towards its substrate Rab. This review describes the architecture of the TRAPP complexes and summarises the mechanisms that underlie their specificity.

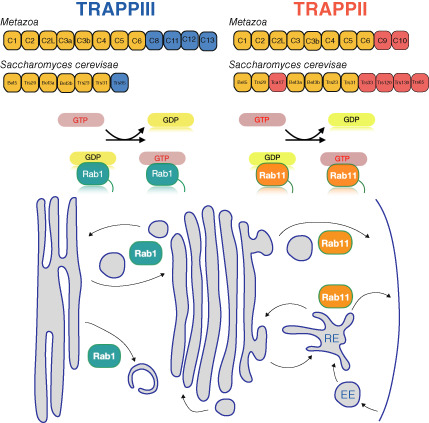

## Abbreviations

**COP**, coat protein complex

**cryo‐EM**, cryo‐electron microscopy

**EMD**, electron microscopy data

**ER**, endoplasmic reticulum

**GAP**, GTPase‐activating protein

**GDI**, GDP dissociation inhibitor

**GDP**, guanosine‐5′‐diphosphate

**GEF**, guanine nucleotide exchange factor

**GTP**, guanosine‐5′‐triphosphate

**HVD**, hypervariable domain

**IgG**, immunoglobulin

**MTC**, multisubunit tethering complex

**PtdIns**, phosphatidylinositol

**TRAPP**, transport protein particle

**TRIPP**, TRAPP‐interacting protein plant

Membrane trafficking ensures that the correct complement of proteins and lipids reaches the appropriate subcellular compartment. The process starts at regions of the donor compartment that deform to generate a transport carrier. The biogenesis of this carrier usually involves the recruitment of coat proteins to the membrane. The resulting vesicle travels to the target membrane, and the initial contact between the two membranes is made by vesicle tethers, which have been divided into two main classes: coiled‐coil proteins and multisubunit tethering complexes (MTCs) [1, 2, 3]. The final traffic event is the fusion of the vesicle to the target membrane driven by the assembly of a set of conserved proteins called SNAREs [soluble NSF‐attachment protein (SNAP) receptors]. The recruitment of the different traffic machinery to their respective membranes is mediated by the activation of small GTP‐binding proteins of the Rab and Arf families [4].

The Rabs and Arfs are molecular switches that are off when GDP is bound and on when GTP is bound. This cycle is controlled by guanine nucleotide exchange factors (GEFs) and GTPase activating proteins (GAPs). The conformational change between these two forms promotes the interaction of the GTP‐bound form with effector proteins. In addition, Rabs are anchored to the membrane by C‐terminal prenyl groups and are extracted from membranes by a chaperone called GDP‐dissociation inhibitor (GDI) that only binds to the Rab in the GDP‐bound state. Guanine nucleotide exchange factors activate each Rab only in the correct location, thus ensuring that this is where the GTP‐bound state accumulates [5, 6, 7].

**Fig. 1:**
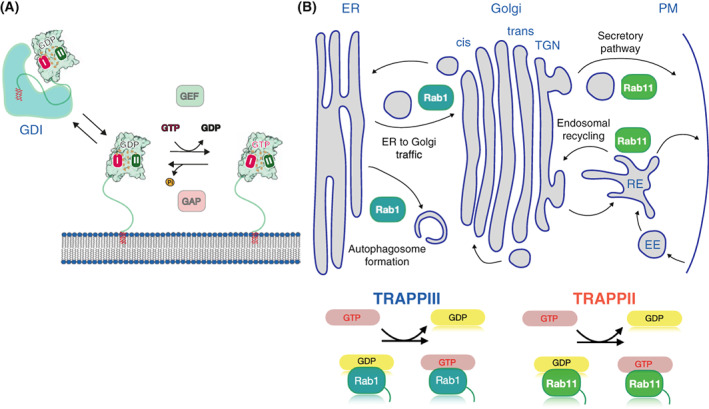
The TRAPP complexes participate in key cellular processes. (A) The Rab activation cycle between GDP to GTP is facilitated by guanine exchange factors (GEFs), and GTPase activating proteins (GAPS). The conformational change between these two forms promotes the interaction of the GTP‐bound form with effector proteins. Rabs are linked to the membrane *via* a flexible C‐terminal hypervariable domain that is prenylated at the C‐terminus. In the cytosol, GDP‐bound Rabs are in a complex with chaperone proteins known as GDP dissociation inhibitors (GDIs), which hide the hydrophobic C‐terminal prenyl groups from the polar environment of the cytosol. (B) Rab1 and Rab11 are master regulators of different steps of the secretory pathway, and Rab1 also regulates autophagy. TRAPPII activates Rab11 whilst TRAPPIII acts on Rab1.

A TRAPP complex was first discovered in yeast *via* the investigation of a subunit that had been identified in genetic screens for components of the secretory pathway, and hence it was named transport protein particle (TRAPP) [8]. Initially, biochemical assays indicated that it was a GEF for the yeast Rab1 ortholog, Ypt1, but results in membrane traffic reconstitution assays, and their interaction with the coat proteins of COPII vesicles, suggested that it might also act as a vesicle tether [9, 10]. In addition, GEF activity for the yeast Rab11 orthologs Ypt31/32 was found associated with TRAPP subunits, leading to the discovery that there are actually two TRAPP complexes that share some subunits but differ in activity. These complexes are highly conserved and, in all organisms examined to date there are two versions: TRAPPII and TRAPPIII. TRAPPIII activates Rab1, a master regulator of the early secretory pathway and autophagy, whilst TRAPPII primarily activates Rab11, which regulates endocytic recycling, exocytosis, and membrane delivery during cytokinesis and ciliogenesis (Fig. 1). Thus, these complexes play essential roles in key cellular processes, and they are associated with a panoply of genetic conditions, known as ‘TRAPPopathies’, that includes neurodevelopmental disorders, muscular dystrophies and skeletal dysplasias [11].

However, there remains some controversy about the GEF activity of the TRAPP complexes towards different Rabs GTPases. In addition, their role as bona fide tethers and their involvement in other cellular processes are still matters of debate. In this review, we will mainly discuss the structures of the TRAPP complexes with a focus on recent findings that explain their Rab specificity. Crystallographic studies had revealed the organisation of the TRAPP core and the mechanisms of nucleotide exchange, while recent single‐particle cryo‐EM studies have shed light on how the specific subunits confer the unique properties of TRAPPII and TRAPPIII.

## The substrates of the TRAPP complex GEFs

Since the discovery of the first TRAPP subunits [8, 12], their function has been proposed to be the activation of Rab1, a GTPase with a key role in both ER‐Golgi traffic and autophagy [13]. Rab1 plays multiple roles in trafficking, recruiting distinct tethers to both COPII and COPI vesicles. Rab1 binds to the coiled‐coil tethers Golgin‐84 and GM130 [14, 15, 16], and to p115 [17], all of which are involved in the maintenance of the Golgi traffic and structure. This GTPase is also essential for the formation of the autophagosome. Rab1 recruits the Atg1 kinase and the tether Atg11 to the pre‐autophagosomal structure, activates HRR25/casein kinase 1δ, and binds to Vps34 complex I, which in turn generates PtdIns(3)P to regulate autophagosome formation [18, 19, 20, 21]. To date, TRAPPIII is the only physiological Rab1‐GEF identified, although the *Legionella pneumophila* protein DrrA has been reported to have nucleotide exchange activity towards Rab1, using a mechanism that is unrelated to that of TRAPPPIII [22].

Evidence that TRAPPII has nucleotide exchange activity towards Rab11 was reported following the finding that the TRAPPIII has activity towards Rab1 [23, 24]. Rab11 localises to the TGN and post‐Golgi structures where it functions in secretion and traffic through recycling endosomes, and it also participates in the delivery of vesicles to the midbody during cytokinesis and several other cellular processes such as ciliogenesis and cell migration [25, 26, 27]. In metazoans, there is another Rab11 GEF, SH3BP5 [28], a V‐shaped coiled‐coil protein, which is not related to the TRAPPs [29, 30]. Partial redundancy between SH3BP5 and TRAPPII provides an explanation for why the TRAPPII‐specific subunits are not essential in metazoans, unlike in yeast [31].

The TRAPP complexes have also been reported to be GEFs for other Rab1‐ and Rab11‐related Rabs. *In vitro*, TRAPPIII has shown GEF activity on Rab43, which belong to the Rab1 family [32], while TRAPPII has been reported to also activate Rab1, and the Rab1 family relatives, Rab19 and Rab43 [33]. Guanine nucleotide exchange factors can be promiscuous *in vitro*, and so determining the physiological importance of these activities will require further investigation.

## The architecture of the TRAPP complexes

**Fig. 2:**
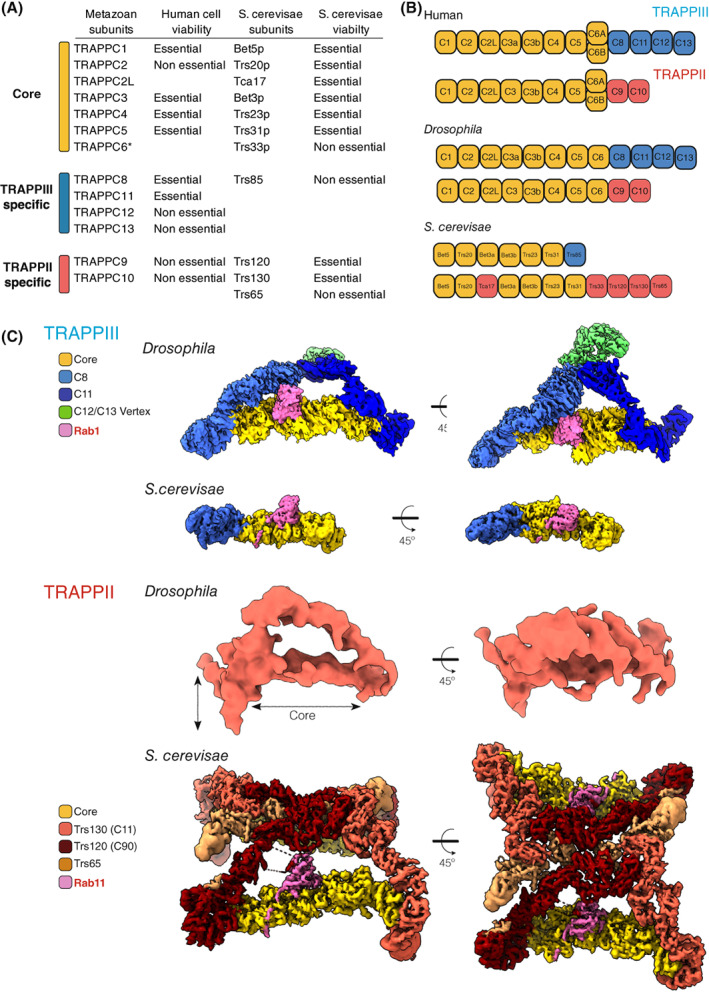
Architecture of the metazoan and yeast TRAPP complexes. (A) The TRAPP subunits. In vertebrates, TRAPPC6 has two isoforms (6A and 6B). Also shown is essentiality for human cell lines, and yeast viability [36, 75]. (B) Subunit composition of the TRAPP complexes. (C) Cryo‐EM density maps for TRAPP complexes from *Saccharomyces cerevisiae* and *Drosophila*, coloured to highlight the TRAPP‐specific subunits. *Drosophila* TRAPPIII (EMD‐12056, with Rab1 (pink) modelled into the map) and TRAPPII (EMD‐12066). *Saccharomyces cerevisiae* TRAPPIII (EMD‐22928) and TRAPPII (EMD‐26270).

The two TRAPP complexes share a common set of seven small subunits, the TRAPP core. This core is sufficient to activate Rab1 *in vitro* [31, 34]. In most species, including metazoans, plants and many fungi, TRAPPIII has four additional subunits that are not present in TRAPPII: TRAPPC8, TRAPPC11, TRAPPC12 and TRAPPC13 (Fig. 2). The former two are essential in human cells and *Drosophila* for Rab1 recruitment to the membrane and thus cell viability, indicating that the core is not sufficient to correctly activate Rab1 *in vivo* [31, 35, 36, 37, 38]. TRAPPII has two additional subunits, TRAPPC9 and TRAPPC10, that are required for Rab11 activation *in vitro* and *in vivo* [31, 39]. All these specific subunits are widely conserved, but some budding yeasts, including the heavily studied *Saccharomyces cerevisiae* (hereafter referred to as ‘yeast’), lack the TRAPPIII subunits TRAPPC11, TRAPPC12 and TRAPPC13 (Fig. 2A).

### The TRAPP core: seven small subunits

**Fig. 3:**
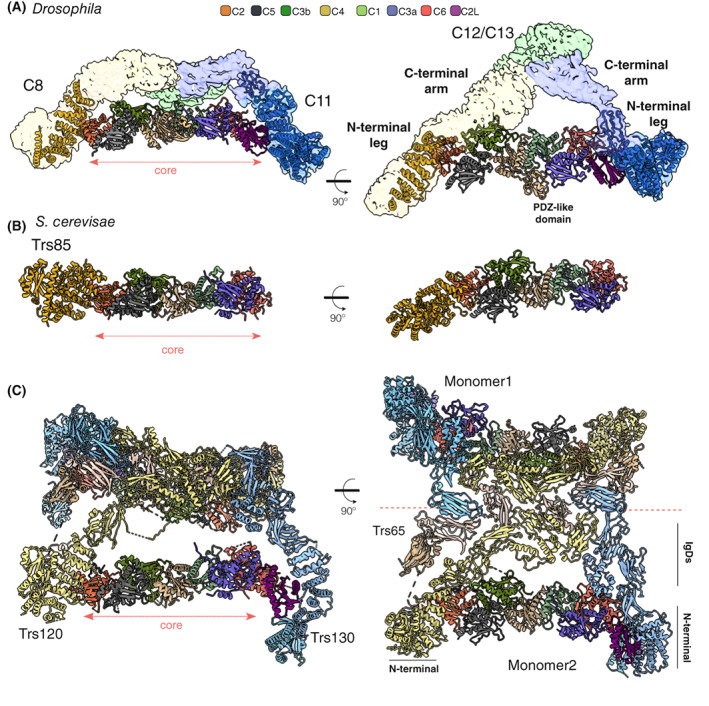
Structural models of the TRAPP complexes. Orthogonal views of the 3D models of *Drosophila melanogaster* TRAPPIII (7B6R), *Saccharomyces cerevisiae* TRAPPIII (7KMT), and *S. cerevisiae* TRAPPII (7U05). The subunits are depicted as ribbons, with the colour code for the core subunits applicable to all three models. (A and B) TRAPPIII‐specific subunits TRAPPC8 (N‐terminal model) and Trs85 (C8) are yellow, TRAPPC11 (N‐terminal model) is blue. (C) TRAPPII‐specific subunits Trs120 (C9) and Trs130 (C10) are light yellow and light blue, respectively. Trs65 is beige.

The assembly of the seven small subunits, TRAPPC1‐C7, forms an octamer with TRAPPC3 present twice (Fig. 2B). Crystallographic studies have revealed the structures of several of the subunits [40, 41, 42, 43, 44, 45], and of different subcomplexes [34, 46, 47, 48]. The combination of these models with the recent cryo‐EM data [49, 50, 51, 52, 53] has revealed the architecture of this octamer: an elongated rod with two flattened surfaces (Fig. 2C). The TRAPPC1 and TRAPPC4 subunits form the centre of this rod and most of the canonical catalytic site for activating either Rab1 or Rab11, and they are flanked on both sides by a TRAPPC3 subunit (C3a and C3b; Fig. 3). The 3D folds of TRAPPC1 and TRAPPC4 are similar, with both comprising a longin domain, a domain found in several other Rab GEFs [34, 48, 54]. Nonetheless, in metazoans, there is a clear difference between TRAPPC1 and TRAPPC4 due to the presence in the latter of a PDZ‐like domain protruding from one side of the rod. Thus, the surface of interaction between C1 and C4 with the C3 subunits is similar, but in the case of C4 and C3, there is an additional region of interaction involving the PDZ‐like domain. The lack of this domain in C1 leaves an open groove on the opposite side of the rod, which the C‐terminal region of C3b can partially occupy. Interestingly, the TRAPPC4 PDZ‐like domain is missing in its yeast counterpart, and so it might be involved in additional regulation or recruitment of the mammalian TRAPP complexes.

On one side of the central tetramer is TRAPPC5 which interacts with C3 and C4, while on the other side is TRAPPC6 which contacts C1 and the other C3 subunit. TRAPPC5 and C6 fold into a so‐called ‘TRAPP domain’ that appears to have evolved in archaea and is conserved into eukaryotes [42, 55]. Finally, TRAPPC2 and TRAPPC2L, two longin‐like proteins, are found at the two ends and are needed to connect to the specific subunits of TRAPPII and TRAPPIII [56, 57, 58, 59, 60, 61] (Fig. 3).

Apart from the two C3 subunits, C1, C5 and C2 are related to C4, C6 and C2L, respectively, so the octamer has a two‐fold rotational symmetry, suggesting that it evolved by gene duplications adding, or altering, one half of the octamer. The most significant divergence among the pairs of related subunits is found between C5 and C6. TRAPPC5 contains a disordered N‐terminal region and an extra C‐terminal α‐helix that is not present in TRAPPC6 [51, 62]. This difference relates to the role of TRAPPC5 in accommodating the hypervariable domain of Rab1 or Rab11, as discussed below.

The initial studies in yeast reported a third complex called TRAPPI, which is a hexameric complex formed by the subunits TRAPPC1 (Bet5), TRAPPC2 (Trs20), TRAPPC4 (Trs23), TRAPPC5 (Trs31) and two copies of TRAPPC3 (Bet3) [10] (Fig. 2B). It has exchange activity on Ypt1 *in vitro*, and indeed it contains the minimal four subunits required for GEF activity. However, a recent report concluded that TRAPPI is an *in vitro* artefact resulting from the intrinsic instability of the complexes, and hence it is TRAPPIII which activates Rab1 in the secretory pathway and autophagy [63]. This proposal is consistent with gel filtration analyses of native TRAPP complexes [61, 64]. The levels of TRAPPI detected in these assays were low, but increased in samples from null mutants lacking the specific subunits or non‐essential core subunits. Furthermore, in filamentous fungi, the TRAPP complexes resemble those of metazoans, and they also appear to lack TRAPPI [61, 65].

### The TRAPP‐specific subunits: comparison of the TRAPPII and TRAPPIII complexes

Unlike the TRAPP core subunits, the structure of none of the TRAPP‐specific subunits was known until recently. In many species, TRAPPIII includes four specific subunits: TRAPPC8, C11, C12 and C13, whereas yeast only has TRAPPC8, and this is a truncated version compared with the metazoan homolog. The first data came from low‐resolution images of yeast TRAPPIII obtained with negative stain EM [50]. They showed that Trs85 (C8), the yeast orthologue of TRAPPC8, is attached to one end of the core *via* the orthologue of TRAPPC2, but no structural details could be seen. However, structural insight into TRAPPIII has now come from single‐particle cryo‐EM studies of the *Drosophila* and yeast complexes [51, 52].

#### TRAPPIII‐specific subunits

The *Drosophila* TRAPPIII cryo‐EM data revealed that the complex has a triangular shape (Fig. 2C). The cryo‐EM map, combined with cross‐linking mass spectrometry, showed that the N‐terminal parts of the large subunits TRAPPC8 and C11 bind to the TRAPP core through C2 and C2L, while the C‐terminal halves of these two subunits extend to a vertex where TRAPPC12 and C13 are located (Fig. 3A). The N‐terminal region of TRAPPC8 (335–660) is an α‐solenoid of 13 α‐helices that includes the region of interaction with C2. Similarly, an α‐solenoid is found in TRAPPC11, with residues 181–566 folded into 15 α‐helices that form the region of interaction with C2L. The mechanism of interaction of TRAPPC8 and C11 with TRAPPC2 and C2L is similar. These large subunits expose two α‐helices of their α‐solenoids to the α‐helix 1 and the loop between β‐strands 1 and 2 of TRAPPC2 and C2L [51].

The C‐terminal parts of TRAPPC8 and C11 are less well resolved, and there is no 3D model based on experimental data, but they form the arms that connect to the TRAPPC12 and C13 vertex. There are two additional points of interaction between the arms and the core in the middle regions of TRAPPC8 and C11 subunits. TRAPPC8 links to a density formed by the connecting loop of the first two α‐helices of TRAPPC3b and C5, while C11 connects to a region formed by the first two α‐helices of TRAPPC3a and C6. This finding is reinforced by the cross‐links between the TRAPPC3 subunits and C8 and C11 [51].

The C‐terminal region of TRAPPC8 is not present in its yeast orthologue Trs85 (C8), consistent with yeast TRAPPCIII lacking TRAPPC11, C12 and C13 that form the rest of the triangle. Overall, the N‐terminal region of the *Drosophila* TRAPPC8 map matches the yeast Trs85 (C8) map. The cryo‐EM structure also reveals that the N‐terminus part of Trs85 (C8), which is not resolved in *Drosophila* TRAPPC8, adopts a GTPase‐like domain, while the C‐terminal region folds into an α‐solenoid comprising five pairs of α‐helices, which correspond to the first 10 α‐helices found in *Drosophila* C8 (Fig. 3B). As in TRAPPC8, the Trs85 (C8) α‐solenoid connects to the TRAPP core by binding Trs20, the yeast TRAPPC2. However, there is a second subsidiary interaction with Trs31, the yeast TRAPPC5 homologue. This interaction is mediated by a loop in the α‐solenoid, an insertion specific to yeast. The function of this interaction is to stabilise the binding to the core [52], presumably because yeast TRAPP has a truncated TRAPPC8, and no TRAPPC11, and so lacks the additional interactions that hold TRAPPC8 into the metazoan complex.

Another feature of Trs85 (C8) is the presence of an amphipathic helix between the GTPase‐like fold and the α‐solenoid. This helix could not be modelled, but it was shown to be required for TRAPPIII membrane binding and Rab1 activation [52]. A similar mechanism to recruit TRAPPIII to membranes might occur with the *Drosophila* complex as amphipathic helices are present in the N‐terminal region of fly TRAPPC8 and C11. However, in the current model, they are arranged with their hydrophobic sides inside the structure [51], which raises the possibility that these helices could be arranged differently in the presence of membranes.

#### TRAPPII‐specific subunits

The sequences of the two specific subunits of the TRAPPII complex, TRAPPC9 and TRAPPC10, are distantly related to those of TRAPPC8 and TRAPPC11, respectively [31, 38, 66]. These pairs of subunits are thus likely to have arisen by gene duplication in a eukaryotic precursor, which is reflected in the low‐resolution 3D map of metazoan TRAPPII having a similar overall architecture to that of TRAPPIII, with the TRAPP core attached to two arms [32, 51]. These arms connect at their opposite ends to form an irregular triangle (Fig. 2C). TRAPPC9 is linked to the core through C2, and C10 is linked *via* C2L, and the pattern of cross‐linking among the core subunits is similar to that found in TRAPPIII [51]. This is consistent with previous low‐resolution EM studies in yeast [49], and recently two independent publications have reported a high‐resolution cryo‐EM structure of the whole TRAPPII complex from yeast, including the structure of a TRAPPII‐Ypt32 (yeast Rab11) activation intermediate [53, 62]. Unlike metazoan TRAPPII, the yeast complex is a dimer, with each monomer resembling the fly TRAPPII triangle. The two specific subunits, Trs120, the TRAPPC9 orthologue, and Trs130, the TRAPPC10 orthologue, hold the TRAPP core, and the dimerisation involves an additional subunit, Trs65 (Fig. 2C). This subunit is not essential and seems to have evolved independently in fungi (see below). The top view of the dimer resembles a butterfly, with the central axis outlined by the extensive interaction of Trs65 with Trs120 (C9) and Trs130 (C10). The TRAPP core of each monomer is positioned parallel to this axis, and, viewed from the ‘edge’, TRAPPII is shaped like an arch. A notable feature of Trs130 (C10) is a ‘leg’ at the most N‐terminal region of the protein that extends below the core of the complex. Comparison of this region with the model of the fly TRAPPC11, has led to the suggestion that the leg of TRAPPC11 is bent relative to the straight leg of Trs130 (C10) [62].

The arrangement and structures of the TRAPP core subunits in TRAPPII are identical to those in the TRAPPIII complex. The Trs33 subunit (yeast TRAPPC6) and the Tca17 subunit (yeast TRAPPC2L) link Trs130 (C10) to the core, whilst Trs120 (C9) connects to the core by binding to Trs20 (C2) at a similar surface to that of Trs85 (C8). The N‐terminal regions of the Trs120 (C9) and Trs130 (C10) subunits resemble the structures of TRAPPC8/Trs85 and TRAPPC11, respectively. They contain a GTPase‐like domain at the N‐terminus followed by an α‐solenoid, which is the site of interaction between Trs130 (C10) and Tca17 (C2L) and Trs120 (C9) and Trs20 (C2). The C‐terminal region of both large subunits comprises four immunoglobulin (Ig)‐like domains (IgDs), which establish a web of interactions with the other three IgDs that form the structure of Trs65 and also hold the two copies of the complex together [53, 62] (Fig. 3C).

Trs65 was initially proposed to be a yeast orthologue of TRAPPC13, but it now appears to be a paralogue that arose by gene duplication in fungi, with some budding yeast then losing TRAPPC13 itself, along with TRAPPC12 and TRAPPC11 [31, 61, 64]. The finding of TRAPPC13 relatives in plants (TRIPP) and vertebrates (C7orf43/TRAPPC14) that are associated with TRAPPII suggests that something similar has occurred in other phyla. TRIPP and TRAPPC14 are both dispensable for TRAPPII integrity, and their interaction with the complex depends on TRAPPC9 and C10 [67, 68]. However, they do not participate in a putative dimerisation of the complex. It appears that vertex formed by the TRAPP arms is a region prone to bind additional subunits that are part of the Trs65/TRAPPC13 family. Structural models predicted by AlphaFold2 indicate that TRAPPC13 is likely to be similar to Trs65 with three IgDs [69]. Two of these IgDs interact with TRAPPC12, which is formed by five β‐catenin‐like armadillo repeats, each of which comprises three α‐helices folded around a central axis (A. Galindo unpublished observation).

## How do the TRAPP complexes function as GEFs?

**Fig. 4:**
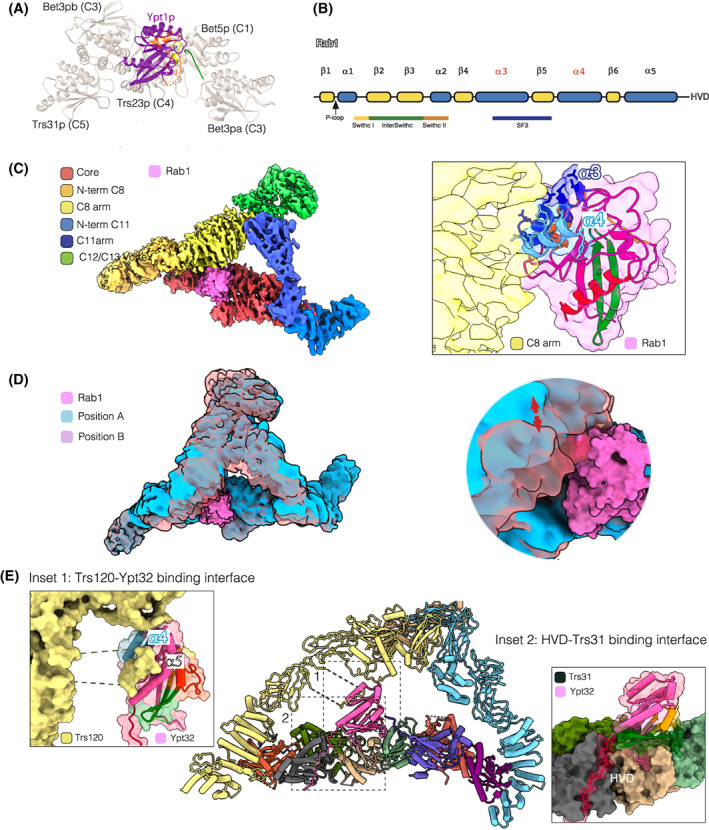
The specific subunits as nucleotide exchange regulators. (A) Ribbon model of the TRAPP core‐Ypt1 interface [88]. Ypt1 is purple, except for switch regions I and II (orange) and the P loop (yellow). The C‐terminus of Bet3a (C3a) is highlighted in green. (B) The secondary structure of Rab GTPases. (C) Rab1 modelled into *Drosophila* TRAPPIII. The inset shows the proximity between TRAPPC8 (yellow) and the α‐helices 3 (dark blue) and 4 (light blue) of Rab1. The Rab1 switch region I is yellow, interswitch green, switch region II orange, and α‐helix 5 red. (D) Movement of TRAPPC8 and TRAPPC11 relative to the core. Superposition of the two extreme positions of the particles from cryo‐EM analysis. (E) The TRAPPII‐Ypt32 complex (7E8T). (Bet5 (C1): light green, Trs20 (C2): orange, Tca17 (C2L): magenta, Bet3a (C3a): purple, Bet3b (C3b): dark green, Trs23 (C4): light brown, Trs31 (C5): grey, Trs33 (C6): red, Trs120 (C9): light yellow, Trs130 (C11): light blue, Ypt32: pink). Inset 1: Surface representation of the Trs120 (C9) IgD1 loop pushing the Ypt32 α‐helices 4 (dark blue) and 5 (light blue). Inset 2: Surface representation of the TRAPP core‐Ypt32 interface. The HVD (red) lies in the Trs31 (C5) pocket.

The structure of most small GTPases comprises a central six‐stranded β‐sheet surrounded by 5 α‐helices (Fig. 4B). The most significant conserved motifs in this structure are the phosphate‐binding loop (P‐loop), which binds the β‐ and γ‐phosphates of nucleotides, and the switch I and II regions that adopt different conformations depending on whether GDP or GTP is bound, and which are crucial for the interaction of the Rabs with both their effectors and also their regulatory GEFs. Rabs also have a hypervariable domain (HVD) at their C‐termini, which is modified with one or two geranylgeranyl groups at the carboxyl end to allow reversible membrane association [70, 71].

### The TRAPP complexes use the same catalytic site for Rab1 and Rab11 nucleotide exchange

Crystallographic studies have revealed that the region of interaction between the TRAPP core and Ypt1, yeast Rab1, is formed by Trs23 (C4), Bet5 (C1), and one of the Bet3 (C3) subunits. However, this subcomplex has no GEF activity, but the addition to the complex of Trs31 (C5) accelerates the dissociation of GDP from Ypt1 more than 400‐fold [34, 48, 72]. Interestingly, the Ypt1‐binding interface is formed by Trs23 (C4) subunits with smaller contributions from the two copies of Bet3 (C3) and Bet5 (C1), but there is no direct interaction between Ypt1 and Trs31 (C5). The TRAPP core interacts primarily with residues in the central β‐sheet of Ypt1, the switch I and II regions and the P‐loop [48]. The C‐terminus of one of the Bet3 (C3) subunits would clash sterically with the switch I region when the Rab is in the nucleotide‐bound form (Fig. 4A). Thus, it favours the opening of the nucleotide‐binding pocket formed by the switch regions, and the transition to the nucleotide‐free TRAPP‐Ypt1 complex. The Bet3 (C3) C‐terminus is not a rigid structural element and may be poorly ordered without Ypt1. Comparing the TRAPP core with the complex of the TRAPP core and Ypt1 shows that the structures differ significantly at the three‐way interface between Trs23 (C4), Trs31 (C5) and Bet3b (C3b). Therefore, the binding of the GTPase depends on conformational changes in that interface, and hence Trs31 (C5) likely functions allosterically by affecting the conformation of Trs23 (C4) [48].

Over the years, there has been debate about the Rab specificity of TRAPPIII and TRAPPII. *In vitro* reconstitution assays have established firmly that TRAPPIII activates Rab1 while TRAPPII primarily has activity on Rab11, but can also act on Rab1 and Rab1 family GTPases to some extent *in vitro* [24, 31, 33, 65, 73]. Using mutations in the catalytic site that prevent the activation of Ypt1, it was found that TRAPPII requires the same residues to activate Ypt31/32, the yeast Rab11 orthologues, [73]. Hence, the TRAPPs represent a noteworthy case of active site plasticity, but it raises the question of how Rab specificity is achieved.

## Determinants of Rab specificity

The fact that both complexes have the same catalytic site and yet activate different Rabs suggests that the specific subunits mediate substrate specificity. It now appears that the specific activation of Rab1and Rab11 depends on a combination of factors that involves the regulatory function of the specific subunits, the distinct features of the particular Rabs and the arrangement of TRAPP complexes on the membrane.

### TRAPPC8 and TRAPPC9 as regulators of GDP to GTP exchange

As mentioned above, the TRAPPIII subunit TRAPPC8 and the TRAPPII subunit TRAPPC9 are related. The TRAPPC8 model, derived from cryo‐EM data [51] and AlphaFold2 prediction, is similar to the reported structure for Trs120 (C9), with the N‐terminal α‐solenoid followed by four IgG domains [53, 62]. In contrast, the fungal Trs85 (C8) lacks the C‐terminal 600–700 residues present in metazoan and plant TRAPPC8. Trs85 (C8) contributes to recruiting yeast TRAPPIII to membranes, *via* an amphipathic helix [52]. Mutants lacking Trs85 (C8) are viable, grow normally and show minimal trafficking defects, although Ypt1 is partially mislocalised and there are defects in autophagy [62, 74]. However, it is not the case with TRAPPC8, which is essential in both flies and mammalian cultured cell lines [31, 75].

There is no complete cryo‐EM model for the metazoan TRAPPIII‐Rab1 complex, but using the crystallographic data to model Rab1 onto the TRAPP core shows that the TRAPPC8 arm would be in close contact with Rab1 through α‐helices α3 and α4 of the canonical Rab structure (Fig. 4C). Interestingly, one of these helices contains one of the Rab subfamily‐specific sequences, RabSF3, that were defined as being conserved between Rabs of the same family but divergent between families [51, 76, 77]. This putative interaction between TRAPPC8 and Rab1 could stabilise the binding between Rab1 and the TRAPP core and thus contribute to specificity. Indeed, the TRAPPIII complex shows significantly more exchange activity on Rab1 than does the TRAPP core alone, and alignments of this region between Rab1 and Rab11 proteins show that many of the Rab1 residues that are predicted to be exposed to TRAPPC8 have a different charge or polarity in Rab11.

The proposal of a specific subunit interacting with a surface located away from the Rab switch regions also arose from the yeast TRAPPII structure. Two independent groups have found three ordered loops of Trs120 (C9) that contact Rab11 [52, 53]. Unlike Trs85 (C8), Trs120 (C9) is more similar to its metazoan counterpart TRAPPC9, and like TRAPPC8 in the fly TRAPPIII structure it serves as a ‘lid’ to enclose the active site, creating an active site chamber. Comparison between Ypt32 in the GDP‐bound state [78] and the TRAPPII‐Ypt32 complex shows a steric clash between Trs120 (C9) and helices α4 and α5 of Ypt32 in the GDP‐bound state. The rearrangement of these helices to overcome the steric hindrance may aid in the rearrangement of the nucleotide binding site and hence nucleotide exchange [53] (Fig. 4E).

In summary, the extended arms of the specific subunits of each TRAPP complex appear likely to interact with the corresponding Rab, and thus determine which Rab undergoes nucleotide exchange *via* the active site formed by the central subunits of the TRAPP core.

### Conformational changes may stabilise the interaction between the TRAPP complexes and their substrate Rabs

The cryo‐EM studies revealed that the TRAPP complexes have intrinsic flexibility that results in conformational changes. These different conformations could affect their GEF activity. In the case of *Drosophila* TRAPPIII, analysis of the particles in the cryo‐EM samples shows a rotational movement of the arms relative to the core [51]. The arms have sufficient flexibility for TRAPPC8 to move over the Rab1‐binding site, and so they could block the binding of the GTPase to the catalytic site on the core. This provides a possible mechanism to regulate exchange activity. We have observed contacts between both arms and the TRAPPC3, C5 and C6 subunits, which raises the possibility that the arm movement triggers an allosteric change linked to the accessibility to the active site [51].

In the case of the dimeric TRAPPII particles from yeast, the individual monomers exhibited two significant conformations which have been referred to as the ‘open’ and ‘closed’ states [53, 62]. Similar to TRAPPIII, the difference between the open and the closed conformation lies in the relative position of the specific subunits and the TRAPP core. Comparing the two conformations, the core in the closed conformation rotates towards Trs120 (C9), so the space enclosed between the core and the Trs120 (C9) arm is smaller than in the open conformation. TRAPPII monomers that are bound to Rab11 only adopt the closed conformation [53, 62], which is consistent with the closing of the Trs120 (C9) ‘lid’ over the TRAPP core is required for stabilising GTPase binding and nucleotide exchange [62]. Interestingly, these conformational changes lead to three different ‘states’ of the whole dimeric TRAPPII complex: state I, with both monomers in an open conformation; state II, with one of them open and the other closed, and state III with both of them closed [53]. It seems most likely that Rab activation by exchange of GDP for GTP always occurs on membranes [6, 71]. Thus, the dimerisation of the yeast TRAPPII particles may increase the time of residence of the whole dimeric complex on the membrane. After finishing the exchange reaction, an empty TRAPP monomer would remain bound to membranes through the other monomer binding Ypt31/32. This could be a particular physiological adaptation required by fungi, which have evolved a specific Trs65 subunit that is key for dimerisation.

### The Rab1 and Rab11 hypervariable domains

The Rab GTPases show high sequence conservation in the nucleotide‐binding domain and possess conserved C‐terminal cysteines and a C‐terminal motif that facilitates interaction with the Rab escort protein during prenylation [70, 79]. However, they have highly divergent HVDs – for example, Ypt1 and Ypt31 nucleotide‐binding domains share 48% sequence identity, but it drops to 19% in the HVDs [48]. Despite this difference, the Rab1 and Rab11 HVDs alone are insufficient for recognition by TRAPPs, and instead it has been proposed that in yeast, the length of the HVD is the basis for a steric gating mechanism that contributes to the specificity of the TRAPP complexes. Thomas et al. [39] generated HVD chimaeras by exchanging the HVD of Ypt1 with that of Ypt31/32, and tested them in GEF assays in the presence of liposomes. They proposed a mechanism that combines direct recognition of the Ypt31/32 HVD by TRAPPII with the steric exclusion of the shorter HVD of Ypt1, which prevents GEF activity towards Ypt1.

However, the length of the HVD is not the only consideration since TRAPPIII does not recognise or activate wild‐type Ypt31/32, which has a longer HVD. The recent cryo‐EM structure offers an explanation. Bagde et al. [62] described unfavourable repulsive interaction of the core with Rab11 due to a negatively charged surface of Rab11, while Mi et al. report a rearrangement of the loop β2‐β3 of Ypt32 upon binding to TRAPPII that affects a Ser‐Ala‐Leu (SAL) motif in the nucleotide‐binding region and thus facilitates nucleotide release [80, 81]. This conformational change does not happen when Ypt32 binds to the TRAPP core or to TRAPPIII, and the repositioning of the SAL motif is due to the interaction with an IgD1‐loop of Trs120 (C9) [53]. These findings might also apply to *Drosophila*, as the fly TRAPP core has almost no detectable activity towards Rab1 on liposomes [51]. The structural data also shows that the HVDs of Ypt1 and Ypt32 bind to the same pocket in Trs31 (C5) in TRAPPIII and TRAPPII respectively, and the arrangement of the HVDs looks similar in both complexes. As we mentioned above, the highest structural divergence among the pairs of related subunits that make up the core is found between TRAPPC5 and TRAPPC6, presumably due to the role of the former in accommodating the HVD.

**Fig. 5:**
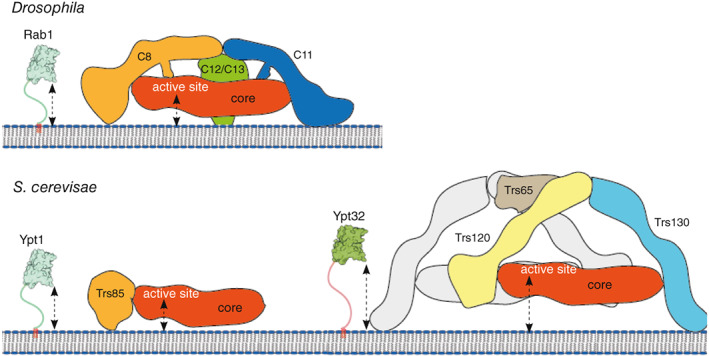
Models of TRAPP complexes on membranes. The C‐terminal hypervariable domain (HVD) of the Rabs stretches from their C‐terminal lipid anchors to the Rab binding site on the TRAPP core. The location of the complexes on the bilayer is hypothetical based on binding sites for the Rabs and their HVDs, and assuming that the N‐terminal regions of the specific subunits mediate membrane contact.

The architectures of the whole TRAPP complexes with their corresponding Rabs, and the fact that activated Rabs are anchored to the membrane *via* the hydrophobic prenyl groups at their C‐terminus, allow prediction of the orientation of these complexes on membranes (Fig. 5). Due to the location of the Rab HVD in the complexes, it seems very likely that the N‐terminal region of the large subunits would be in contact with the membrane. The length of the HVD must span between the active site and the membrane surface. The extended N‐terminal ‘leg’ of Trs130 (C10) could explain the lack of activity of yeast TRAPPII towards Ypt1 when TRAPPII is attached to membranes by binding the GTPase Arf1 [39]. Likewise, in the low‐resolution fly TRAPPII model, there is a leg region that protrudes further than the TRAPPIII's legs, suggesting that it is also responsible for lifting the active site from the membrane.

How the N‐terminal ‘legs’ interact with membranes has been investigated for yeast TRAPPIII, with the Trs85 (C8) amphipathic helix proposed to have role in membrane recruitment [52]. We believe that similar amphipathic helices are probably present in the N‐terminal region of metazoan TRAPPC8 and TRAPPC11 subunits, consistent with the finding that this region of TRAPPC8 is among those that shows altered hydrogen‐deuterium exchange in the presence of liposomes [32]. As for TRAPPII, in yeast, it is recruited to the Golgi membrane through the cooperative action of anionic lipids and activated Arf1 GTPase that binds to the specific subunits [73]. In metazoans, both TRAPPII and TRAPPIII could also contact membranes, or other regulators, through the apex formed by the specific subunits.

## Are the TRAPP complexes vesicle tethers?

The TRAPP complexes are distinct from other GEFs in that their size and subunit number exceeds that typically required for spatial activation of a small GTPase. Each complex is around 500 kDa, and the arrangement of the TRAPP‐specific subunits as long arms that extend to form a triangle with the core provides a large surface area that components of membrane traffic could bind without sterically interfering with GEF activity.

After the discovery of the first TRAPP subunits in yeast, they were initially found to interact genetically with ER‐to‐Golgi SNAREs (Bet1) and Golgi tethering factors such as Uso1p, and this, along with *in vitro* experiments, suggested that TRAPPs are involved in a late‐stage of ER‐to‐Golgi transport [8, 12, 82]. Subsequent studies reported direct interactions with coat proteins, with specific TRAPP subunits apparently differentiating ER‐derived COPII‐coated vesicles from Golgi‐derived COPI‐coated vesicles, leading to the proposal that TRAPP complexes could act as vesicle tethers. There is certainly evidence that other tethering complexes can interact with coat complexes. Mutations in coat subunits, as well as mutations in the corresponding SNAREs lead to the accumulation of large numbers of coated vesicles [83, 84, 85, 86, 87]. These results suggest that, at least in some cases, vesicles could still carry their coat when they arrive at the target membrane, and so coats on vesicles could plausibly be used to tether the vesicle to the target membrane. In the case of the TRAPP complexes, the first reported interaction with a vesicle coat was that between the TRAPP core and the Sec23 subunit of the COPII coat [88]. In the case of TRAPPII, an interaction has been reported with γ‐COP of the COPI coat [89]. TRAPPC9 binds γ‐COP regardless of the presence of TRAPPC10, suggesting TRAPPC9 and γ‐COP directly interact with each other [90]. Similarly, another TRAPPIII specific subunit, TRAPPC12, has also been reported to be at ER exit sites and to interact with the Sec13/Sec31A heterotetramer [91]. However, further work will be required to determine which, if any, of these various interactions is physiologically significant, and precisely what role they play in the cycle of vesicle formation and delivery [92]. In addition, there is no direct evidence for an interaction between the TRAPP complex and SNARES, or Rab GTPases, in contrast to what has been seen with some other vesicle tethers. Thus, the TRAPPs may not be direct vesicle tethers, and instead only serve to activate their substrate Rabs in an intermediate step of vesicle delivery that occurs before the actual tethering step.

## The roles of the TRAPP complexes in cellular functions

TRAPPIII has a key role not only in ER to Golgi traffic but also in autophagy through the activation of Rab1. In yeast, deletion of Trs85 (C9) causes a defect in autophagy, with a similar defect seen with the thermosensitive mutant *ypt1‐1* [74, 93, 94, 95]. Both proteins are needed for selective autophagy during fed conditions, and also for efficient starvation‐induced autophagy, and they have been reported to be localised at the phagophore assembly site. The role of the Trs85 (C9) amphipathic helix in anchoring the TRAPP core to the membrane surface is required both for efficient secretion and for autophagy [52]. Atg9 is a key factor in the initiation of autophagy, and the current view is that Atg9‐containing vesicles transport the TRAPPIII‐Ypt1 complex to the phagophore assembly site [95], allowing this complex to function in the process of autophagosome formation, recruiting other autophagy factors like Atg1 and Atg11 [18, 19].

In vertebrates, TRAPPC8 interacts with TBC1D14 [37], a member of a family of Rab GAPs that has a role in regulating autophagy, although its Rab substrate is unknown [37, 96]. TRAPPC11 has been reported to interact with ATG2B, a component of autophagy, as well as its binding partner WIPI4, with both thought to be involved in the phospholipid transfer to form the forming autophagosome [97]. It has also been reported that TRAPPC13 is important for autophagic flux under certain stress conditions [98].

TRAPPII and Rab11 regulate endosomal recycling and exit from the Golgi and have also been reported to have specialised roles in cytokinesis and ciliogenesis. For instance, TRAPPII acts *via* Rab11 to target Rabin8, the GEF for Rab8, to the centrosome for primary cilia assembly [68, 99]. Interestingly, TRAPPIII has also been reported to be involved in cilium formation: TRAPPC12 and TRAPPC8 interact sequentially with OFD1, a protein that localises to the centriolar satellites and is critical for cargo entry and exit from the primary cilium [100]. In recycling endosomes, TRAPPII has been reported to bind to the Rho GEF, Kalirin [101], and in Golgi traffic, yeast TRAPPII has been reported to bind Gyp6, the GAP for Ypt6, yeast Rab6 [102], and also the Arf1 exchange factor Gea2 that facilitates COPI vesicle traffic [103]. In addition, immunoprecipitated metazoan TRAPPII has been reported to have GEF activity towards Rab18, although this was not seen with a recombinant form of the complex [31, 33, 90], with TRAPPII proposed to activate Rab18 to regulate lipid droplet homeostasis [90, 104]. Many of these interactions still need further confirmation and determination of physiological relevance, but they could represent crosstalk among GEFs and GAPs, with the TRAPP complexes serving to regulate subsequent trafficking steps.

## Conclusions and perspectives

The roles and composition of the TRAPP complexes have long been controversial topics in the membrane traffic field. These large complexes function as Rab GTPase GEFs, but a role as tethers has also been suggested. Since their discovery almost 25 years ago, they have been primarily characterised in yeast. Recent structural studies have revealed the architecture of yeast TRAPPII and TRAPPIII as well as *Drosophila* TRAPPIII, leading to a better understanding of the specific activation of two master traffic regulators, Rab1 and Rab11. This work has also shed light on the topology of the complexes on the membrane and hence provided clues as to how the TRAPP complexes are orientated on membranes. Key open questions include understanding how they are recruited to the specific membranes on which they function, how they are regulated, and what interactions they make and regulate in addition to activating their substrate GTPases. Although much remains to be understood about the TRAPP complexes and their regulation, the recent structural studies of the complexes provide an excellent foundation on which to expand our knowledge of TRAPP function and to help understand the molecular basis of TRAPP‐associated diseases.
